# Molecular signaling in zebrafish development and the vertebrate phylotypic period

**DOI:** 10.1111/j.1525-142X.2010.00400.x

**Published:** 2010-03

**Authors:** Aurélie Comte, Julien Roux, Marc Robinson-Rechavi

**Affiliations:** a Department of Ecology and Evolution, Biophore, Lausanne UniversityCH-1015 Lausanne, Switzerland; b Swiss Institute of BioinformaticsCH-1015 Lausanne, Switzerland

## Abstract

During development vertebrate embryos pass through a stage where their morphology is most conserved between species, the phylotypic period (approximately the pharyngula). To explain the resistance to evolutionary changes of this period, one hypothesis suggests that it is characterized by a high level of interactions. Based on this hypothesis, we examined protein–protein interactions, signal transduction cascades and miRNAs over the course of zebrafish development, and the conservation of expression of these genes in mouse development. We also investigated the characteristics of genes highly expressed before or during the presumed phylotypic period. We show that while there is a high diversity of interactions during the phylotypic period (protein–DNA, RNA–RNA, cell–cell, and between tissues), which is well conserved with mouse, there is no clear difference with later, more morphologically divergent, stages. We propose that the phylotypic period may rather be the expression at the morphological level of strong conservation of molecular processes earlier in development.

## INTRODUCTION

During the metazoan embryonic development, the complexity of the organism increases from one cell to an integrated multicellular animal. This is accompanied not only by an increasing number of parts, but also by changes in the pattern of interactions among these parts (Raff 1996). In very early development, connections are limited, with the embryo mainly organized along two axes. When organ primordia form, the body becomes partitioned in “modules,” between which numerous interactions take place. At late stages the organs continue to differentiate, but the “modules” are now semi-independent, and the interactions mainly occur within them. This model has been linked to the observation that mid-development is the most morphologically conserved period among vertebrate embryos (Duboule 1994; Richardson 1995; Raff 1996; Galis and Metz 2001; Irmler et al. 2004;), hence the term “phylotypic stage” or “phylotypic period.”

In practice, such interactions must involve molecular pathways of signaling and regulation. Morphological models do not specifically predict that molecular pathways themselves should vary. But if signaling is dramatically different between early, middle (“phylotypic”), and late development, we expect to see changes in the activity of signaling pathways during development. Moreover, if changes in signaling are causal to the phylotypic period, we expect the timing of some changes in signaling to correspond with the boundaries of this period. Characterizing such molecular variation might help to reconcile divergent observations of developmental variation at the morphological and the genomic level (Galis and Metz 2001; Davis et al. 2005; Hazkani-Covo et al. 2005; Irie and Sehara-Fujisawa 2007; Roux and Robinson-Rechavi 2008;).

In this study, we use expression information to relate zebrafish genes to developmental stages, and investigate the variation in protein–protein interactions (PPI), signal transduction cascades, and micro-RNA signaling. We also investigate whether the timing of gene expression is conserved in mouse. This allows us to distinguish signaling pathways which are most active in early, mid, or late development, and can be related to the different phases of morphological integration.

## MATERIALS AND METHODS

### Microarray data and clustering

Microarray data of zebrafish (*Danio rerio*) development were retrieved from ArrayExpress (E-TABM-33; Parkinson et al. 2007). This experiment used an Affymetrix GeneChip Zebrafish Genome Array (A-AFFY-38) with 15,617 probes, which correspond to 8922 Ensembl genes (Hubbard et al. 2007). Fifteen stages, two replicates per time point, were sampled: 15 min, 6, 8, 9, 10, 11.7, 16, 24, 32, 48 h, 4, 5, 14, 30, and 90 days, spanning zygote, gastrula, segmen-tation, pharyngula, hatching, larval, juvenile, and adult stages.

Raw CEL files were normalized using the gcRMA package (Wu et al. 2004) of Bioconductor (Gentleman et al. 2004). We used the “affinities” model of gcRMA, which uses mismatch probes as negative control probes to estimate the nonspecific binding of probe sequences. The normalized values of expression are in log2 scale, which attenuates the effect of outliers.

Presence and absence calls were retrieved from ArrayExpress. The method used for absolute detection of transcripts was the MAS5 algorithm.

For the 1965 Ensembl genes that are represented by more than one probe, we used the mean of all the probe values as the gene expression value, and we considered the gene present if more than half of its probe calls determined it as present.

The two replicates were used for calculations and plotting except for clustering where we used the average of the two replicates. As in Roux and Robinson-Rechavi (2008) we did not consider the first time point of the data (15 min, fertilization).

The genes were separated in 25 clusters (see supporting information Fig. S1) using the fuzzy *c*-means soft clustering algorithm implemented in the Mfuzz package (Futschik and Carlisle 2005) of Bioconductor. From these clusters we formed three groups of genes: highly expressed in early development (cluster 15; 160 “early” genes), highly expressed at the presumed phylotypic period (clusters 1, 20, and 23; 475 “organogenesis” genes), and highly expressed after the presumed phylotypic period (clusters 3 and 8; 412 “late” genes).

### PPI

Human PPI were downloaded from the BioGRID (Stark et al. 2006), IntAct (Hermjakob et al. 2004), and HPRD (Mishra et al. 2006) databases. Interacting proteins were, respectively, mapped from HGNC symbol, Uniprot Accession, and EntrezGene ID to Ensembl human genes. Six hundred and seventy-one EntrezGene IDs that corresponded to more than one Ensembl human gene were removed. The Ensembl human–zebrafish one-to-one orthologs were retrieved from Ensembl. We merged the interaction data of the three databases yielding a dataset of 5277 protein pairs with associated expression data.

For each developmental stage we retained interactions for which both interacting proteins were expressed according to the present/absent calls of the microarray data.

Degree, betweenness, and closeness centrality measures (Freeman 1978/79) were calculated for each interacting protein at each stage using the R igraph package (R Development Core Team 2007; http://www.R-project.org/). Spearman correlation between gene expression and centrality measures was performed for each stage.

### Signal transduction genes

Zebrafish genes and their associated GO IDs were retrieved with Biomart (Kasprzyk et al. 2004) and the GO terms were downloaded from Gene Ontology (Ashburner et al. 2000; November 3, 2008). Genes annotated with GO terms that contained “signal” and “transduction,”“receptor,”“kinase,” or “transcription” were retrieved. This resulted in 421 signal and transduction, 413 receptor, 299 kinase, and 691 transcription genes for which expression data existed; 47 genes were annotated with both “receptor” and “transcription” terms (i.e., nuclear receptors). The number of expressed genes for each stage and each replicate were determined according to the present/absent calls of the microarray data; the mean of the two replicates was used.

**Fig. 3:**
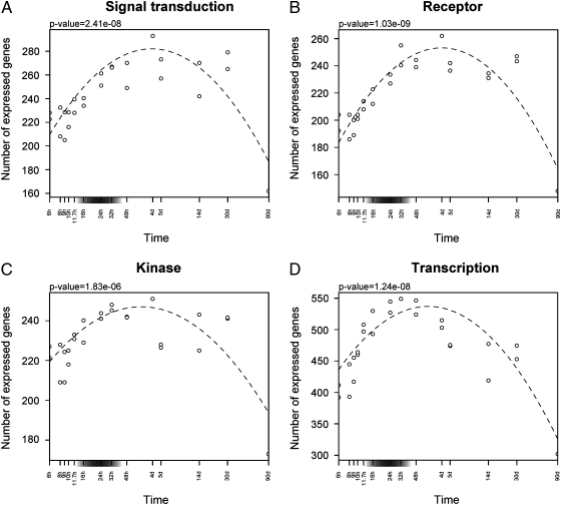
Variation of gene expression for signal transduction genes during development. Number of expressed genes per developmental stage annotated with GO terms containing (A) both “signal” and “transduction,” (B) “receptor,” (C) “kinase,” and (D) “transcription.” A polynomial model was fitted to the data (dashed line parabolas) with *P*-values indicated above each plot. The gray boxes on the *x*-axes indicate the presumed phylotypic period. The *x*-axes are in logarithmic scale.

A linear regression between developmental time and number of expressed genes was fit to the data. To test for an hourglass-like model, we adjusted a parabola (polynomial model of order 2), as in Roux and Robinson-Rechavi (2008). We used an ANOVA to estimate if the increase in fit to the data (*r*) between the linear and parabola models was significant. A Bonferroni correction was applied to correct for multiple testing, considering the seven regressions of Figs. 3 and 7.

### Gene ontology analysis

Over and under representation of GO terms for “early,”“organogenesis,” and “late” genes were tested with a Fisher exact test using the Bioconductor package topGO (Alexa et al. 2006). The reference set was all the Ensembl genes that were represented by a probe on the microarray. The “elim” algorithm of topGO was used, allowing decorrelation of the GO graph structure, reducing nonindependence problems. A False Discovery Rate correction was applied and gene ontology terms with an FDR<5% were reported.

### Phenotypes and localization of expression data

Zebrafish genotypes and phenotypes were recovered from the Zebrafish Information Network (ZFIN; July 2008; Sprague et al. 2006). We selected the phenotypes corresponding to single gene mutants grown in normal conditions and to wild-type lines treated with only one morpholino targeting a single gene. The localization of gene expression for wild-type lines grown in normal conditions was also retrieved from ZFIN. Genes were mapped from ZFIN IDs to Ensembl IDs; 573 ZFIN IDs that correspond to more than one Ensembl ID were removed. There was mutant phenotype information for 22 “early” genes, 29 “organogenesis” genes, and seven “late” genes. And 96 “early” genes, 294 “organogenesis” genes, and 211 “late” genes had localization of expression data.

The significance of the difference between the mean numbers of abnormal phenotypes or structures with expression per gene of the three categories was determined with a Kruskal–Wallis test. When the difference was statistically significant, pairwise Wilcoxon tests were performed; *P*-values were adjusted for multiple testing using the Bonferroni correction.

Enrichment and depletion of expression in anatomical structures (ZFIN) for “early,”“organogenesis,” and “late” genes were tested with a Fisher exact test using a version of the Bioconductor package topGO (Alexa et al. 2006) modified to handle any OBO ontology (Alexa and Roux, unpublished data). The reference set, the algorithm and the FDR value are the same as for the GO analysis. We used only structures that show expression of at least five genes.

### miRNAs targets and expression

Zebrafish miRNAs were downloaded from the miRBase database (Griffiths-Jones et al. 2008).

A time series of miRNA microarray data during zebrafish development (Wienholds et al. 2005) was retrieved (GSE2625) from GEO (Barrett et al. 2007). In this experiment a microarray developed for the detection of mammalian miRNAs was used to measure the expression of zebrafish miRNAs, which is made possible by the very strong sequence conservation of miRNAs. Fifteen stages were sampled: 0, 4, 8, 12, 16, 20, 24, 28, 32, 40, 48, 56, 64 h and 4 days, spanning zygote, blastula, gastrula, segmentation, pharyngula, hatching, and larval stages, as well as male and female adults. Adult time points were removed from our analyses, as their expression value did not correspond to what was reported in Wienholds et al. (2005), even after normalization. Expression data were normalized using the control probes pre-3, pre-4, and pre-5, and subsequently log transformed. Each miRNA is represented by five probes on the microarray. We used the mean of all the probe values as the miRNA expression value. We thus had expression data for 109 zebrafish miRNAs.

The miRNAs were separated in two clusters (Fig. S2) using the fuzzy *c*-means soft clustering algorithm implemented in the Mfuzz package (Futschik and Carlisle 2005) of Bioconductor. We defined the 65 miRNAs from cluster 1 as “early onset” and the 44 miRNAs from cluster 2 as “late onset.”

EIMMo (Gaidatzis et al. 2007) target predictions for zebrafish miRNAs were retrieved from http://www.mirz.unibas.ch/miRNAtargetPredictionBulk.php (v3, January 2009). Targets were mapped from RefSeq IDs to Ensembl zebrafish genes. Ensembl genes that corresponded to more than one RefSeq IDs were removed.

Among the genes for which we have expression data, 119 are targeted only by “early onset” miRNAs and 253 only by “late onset” miRNAs. To assess the significance of the difference between median expression across development of the “early onset” miRNAs targets and the “late onset” miRNAs targets, we used a randomization approach (as in Roux and Robinson-Rechavi 2008). We pooled all the targets, randomly formed two new groups of the same size as the original groups (*n*_1_=119, *n*_2_=253) and calculated the difference in median expression between the two random groups, with 10,000 repetitions.

### Conservation of gene expression in mouse

Expression information (Affymetrix, “high quality”) during development was retrieved for zebrafish (6305 genes) and mouse (*Mus musculus*; 17,192 genes) from Bgee, a database to compare expression data between species (Bastian et al. 2008). The Ensembl mouse–zebrafish one-to-one orthologs were retrieved from Ensembl. Although homologous developmental stages cannot be defined precisely, Bgee implements broadly defined metastages, which can be compared between species. A precise description of the metastages and the correspondence between mouse or zebrafish stages to them can be found in the files stages.obo and stage_association.txt downloadable at http://bgee.unil.ch/bgee/bgee?page=download.

To quantify the conservation of coexpression of interacting proteins over developmental meta-stages, we calculated for each metastage the number of interacting pairs of proteins for which both zebrafish and mouse one-to-one orthologs are expressed. This was compared with the coexpression of random pairs of zebrafish genes (10,000 randomizations). We plot the mean ratios of observed coexpression of PPI pairs to random pairs.

Zebrafish and mouse genes and their associated GO IDs were retrieved with Biomart and the GO terms were downloaded from Gene Ontology (June 25, 2009). Genes annotated with GO terms that contained “signal” and “transduction,”“receptor,”“kinase,” or “transcription” were retrieved. We kept the mouse–zebrafish one-to-one orthologs with GO annotation and expression data in both species. This resulted in 98 pairs for signal transduction, 124 for receptor, 127 for kinase, and 307 for transcription. We calculated the total number of mouse and zebrafish genes of each gene category expressed at each metastage, as well as the number of ortholog pairs both expressed at each metastage. To assess the significance of the number of orthologs expressed, we randomly created pairs of mouse–zebrafish genes from the same gene category. Repeating this process 10,000 times, we could define 1% confidence intervals.

## RESULTS

### Protein interconnectivity is highest in early development

We first examined position in the PPI network, according to timing of expression of the genes encoding the interacting proteins. Proteins at the center of the network are more connected than those at the network periphery. Consequently, determining the network centrality of a protein is equivalent to evaluating its level of connectivity. Of note, we transferred information on human interactions to the zebrafish; whereas this may affect the precision of our results, it is probable that trends are essentially correct (Alexeyenko and Sonnhammer 2009).

**Fig. 1:**
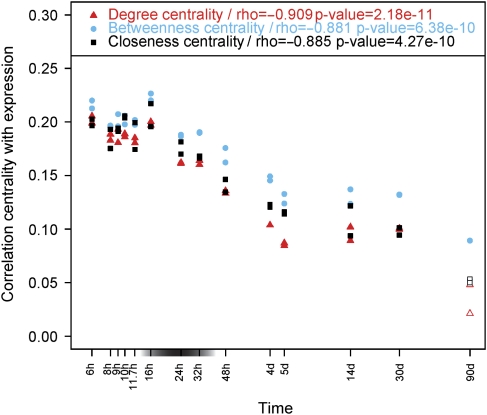
Variation of centrality in the protein–protein interaction network during development. Variation of the correlation between centrality and gene expression level with timing of gene expression across zebrafish development. The three curves represent degree centrality (red triangles), betweenness centrality (blue circles), and closeness centrality (black squares). Filled points indicate a significant correlation with expression at a given stage. Spearman correlations (coefficient rho) were computed between the correlation of centrality and expression, and developmental time. The gray box on the *x*-axis indicates the presumed phylotypic period. The *x*-axis is in logarithmic scale.

We used three different measures to quantify the centrality of proteins: degree, betweenness, and closeness centrality (Freeman 1978/79). Degree centrality is defined as the number of links incident upon a node; it is a local measure. Betweenness and closeness centrality are global measures: the first reflects the number of occurrences of a node on shortest paths between other nodes, whereas the second reflects “shallowness” to other nodes. At each stage we computed Spearman's correlation between these centrality measures and gene expression from microarray data, to remove the possible confounding effect of expression level on studies of connectivity (Pal et al. 2006). The three centrality measures give similar results (Fig. 1). At all stages the correlation is positive, confirming that highly expressed proteins tend to be central and to participate in many interactions. The correlation decreases over developmental time, suggesting that early expression has a higher relation to protein–protein connectivity than late expression. This is coherent with results from Liang and Li (2009), who contrasted the centrality and connectivity of developmental versus nondevelopmental genes. The presumed phylotypic period does not show any specific trend.

**Fig. 2:**
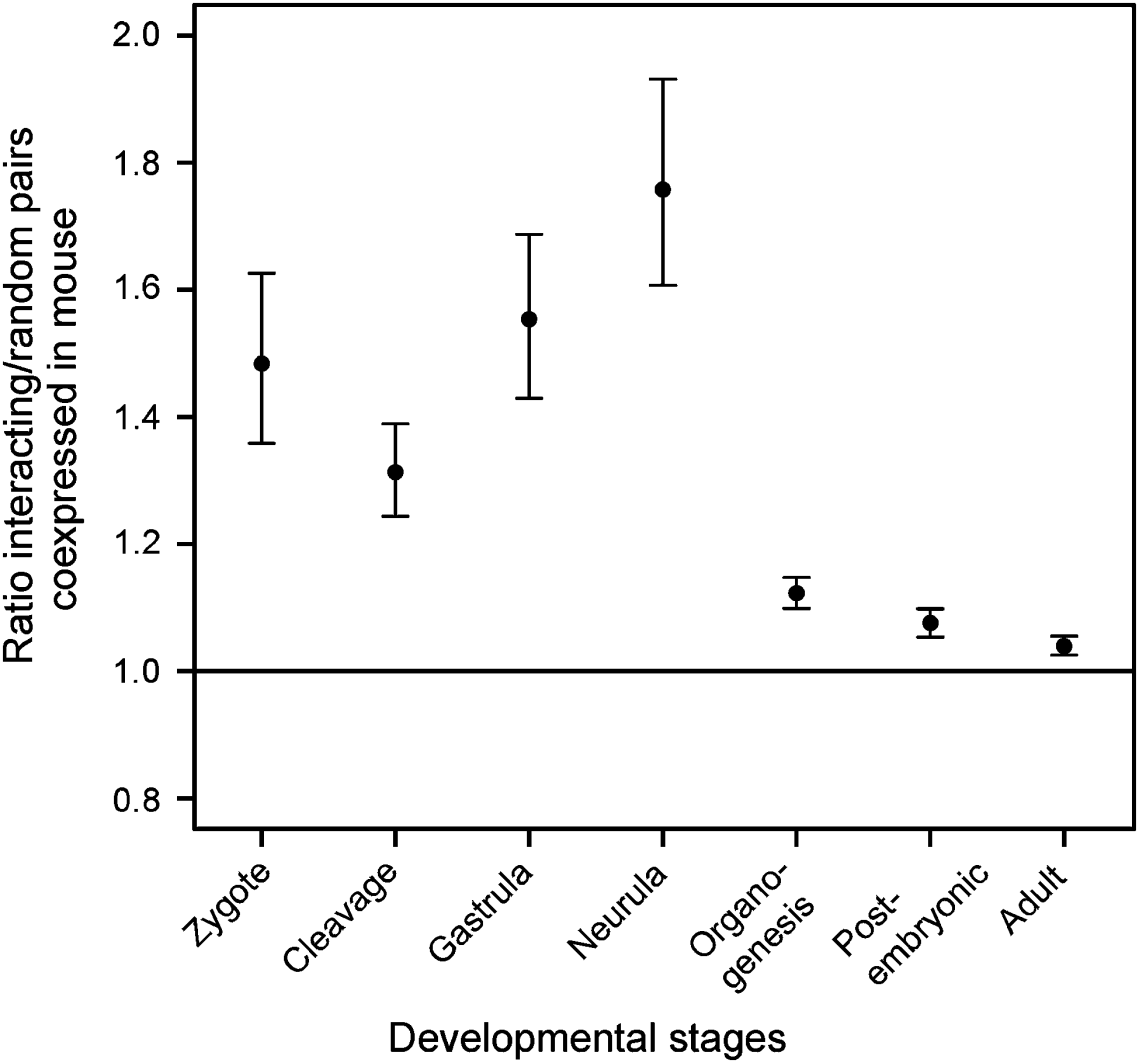
Conservation of coexpression of pairs of interacting proteins between zebrafish and mouse. Mean ratios of the number of pairs of interacting proteins whose coexpression is conserved between zebrafish and mouse at a given developmental meta-stage, to the number of random pairs of proteins whose coexpression is conserved between zebrafish and mouse. Bars represent percentiles of ratios (1% and 99% of repetitions). Organogenesis includes the presumed phylotypic period. The *x*-axis is not proportional to time, as the mapping of the stages of the two species compared on meta-stages is different. The horizontal line indicates a ratio of 1, that is conservation of interacting pairs not different from random pairs.

To verify the evolutionary relevance of these observations, we measured whether the orthologs of pairs of genes, which are both expressed in the same broad developmental stage in zebrafish, are also both expressed in the corresponding stage in mouse. Although genes encoding pairs of interacting proteins have more conservation of coexpression than other genes at all stages, conservation is strongest in early development (zygote–neurula, Fig. 2). In later development, including the phylotypic period (included in organogenesis), the conserved coexpression of interacting proteins is much weaker.

### Signal transduction is highest in the larva

To investigate interactions between cells or tissues, we studied the expression of genes annotated with GO terms containing both “signal” and “transduction,” as well as genes annotated as key components of signaling: receptors, kinases, and transcription genes. Each of these categories individually shares the general pattern of high correlation between PPI centrality and expression level early in development (Figs. S4 and S5).

The number of signal transduction, receptor, and kinase genes expressed increases progressively to reach a maximum at 4 days (larval stage) and then decreases at later stages (Fig. 3, A–C). Excluding photoreceptors from the analysis of receptors, to check for potential bias due to eye development, does not modify observed trends (data not shown). Pairwise comparisons confirm that a significantly higher proportion of genes is expressed at 4 days than at 24 h for signal transduction and receptors (comparison of proportions over both repetitions of the experiment, Bonferroni's correction [5 tests]; signal transduction *P*=0.0011; receptor *P*=0.0080). Transcription genes peak earlier (Fig. 3D), at 32 h, which corresponds to late pharyngula, the stage most often associated with the phylotypic period (Duboule 1994). There are significantly more transcription genes expressed at 24 or 32 h than at 4 days (32 h vs. 4 days: *P*=0.0011). But abundant expression remains during larval development. Genes which possess both transcription and receptor functions (i.e., nuclear receptors) show the same behavior as receptors (data not shown).

**Fig. 4:**
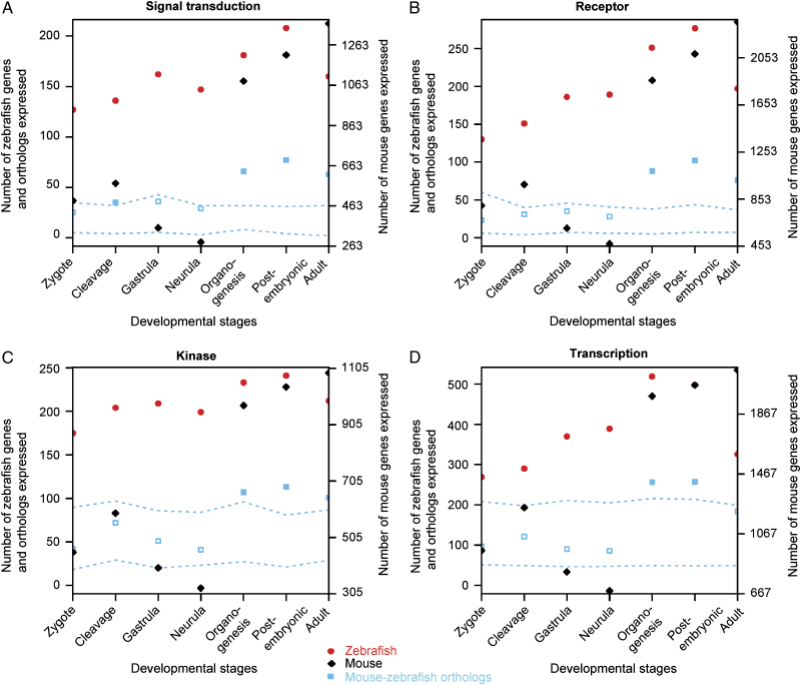
Conservation of gene expression for signal transduction genes between zebrafish and mouse. Number of zebrafish (red circles) and mouse (black diamonds) genes, and ortholog pairs (blue squares) expressed per developmental stage for (A) signal transduction, (B) receptors, (C) kinases, and (D) transcription. The dotted lines represent the 1% confidence interval for conserved expression of orthologs; significant numbers of orthologs expressed are represented by filled squares. Organogenesis includes the presumed phylotypic period. The *x*-axis is not proportional to time, as the mapping of the stages of the two species compared on meta-stages is different. The scale of the *y*-axis is different for mouse, as more data are available.

For all components of signaling tested, the expression of orthologs is significantly conserved in mouse development at all late stages, from organogenesis to adulthood (Fig. 4); but not in early development. There is no specific peak of conservation in organogenesis, which includes the phylotypic stage.

Thus signal transduction appears important, and evolutionarily conserved, over a large period of development, which starts during the phylotypic period but lasts into postembryonic development.

### miRNA expression increases progressively through development

It has been proposed that the control of protein coding genes by miRNAs leads to a gain of developmental precision at the cost of a loss of evolutionary plasticity (Sempere et al. 2006). This suggests that the less morphologically variable developmental stages could be under stronger miRNA control.

**Fig. 5:**
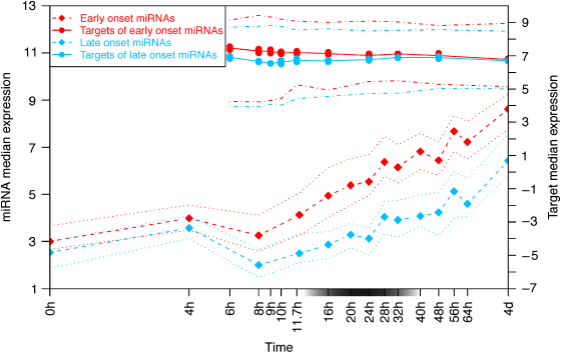
Variation of miRNA and target genes expression during development. Median expression of “early onset” miRNAs (red dashed line, diamonds; *n*=65) and their targets (red line, circles; *n*=119), and of “late onset” miRNAs (blue dashed line, diamonds; *n*=44) and their targets (blue line, circles; *n*=253). Dotted lines represent quartiles of miRNA expression; dot-dashed lines represent quartiles of target gene expression. Differences between the two target groups and significance are show in Fig. S3. The gray box on the *x*-axis indicates the presumed phylotypic period. The *x*-axis is in logarithmic scale.

The expression of miRNAs during zebrafish development (Fig. S2) suggests a classification into two categories: “early onset” miRNAs whose expression starts to increase before the presumed phylotypic period (11.7 h, segmentation), and “late onset” miRNAs whose expression rises later (28 h, pharyngula; Fig. 5). In both groups a peak of expression is detected at 4 h (blastula). It corresponds most probably to the maternal-zygotic transition (Thatcher et al. 2007). No other peak of expression is noticed along development.

Expression of targets of the “late onset” is stable across development, whereas “early onset” targets experience a small decrease during development (Fig. 5, Fig. S3). As miRNAs are negative regulators of gene expression, the observation of a decrease in the expression level of targets of “early onset” miRNAs once these miRNAs are expressed is not surprising. However, the interpretations of this result should be considered with care. The difference in median expression between the targets of the two categories of miRNAs is globally not significant across development, as assessed by a randomization (except for one of the replicates at time point 9 h; Fig. S3). It is probable that by using gene and miRNA expression data from the whole organism, we have missed fine regulation in specific regions of the embryo. It is also possible that the high rate of false positives in databases of target predictions (Alexiou et al. 2009) renders this result less accurate or precise.

There is no comparable data on expression of miRNAs during development of other vertebrate species, so we cannot investigate evolutionary conservation of these patterns.

### Characteristics of genes expressed during different developmental periods

**Fig. 6:**
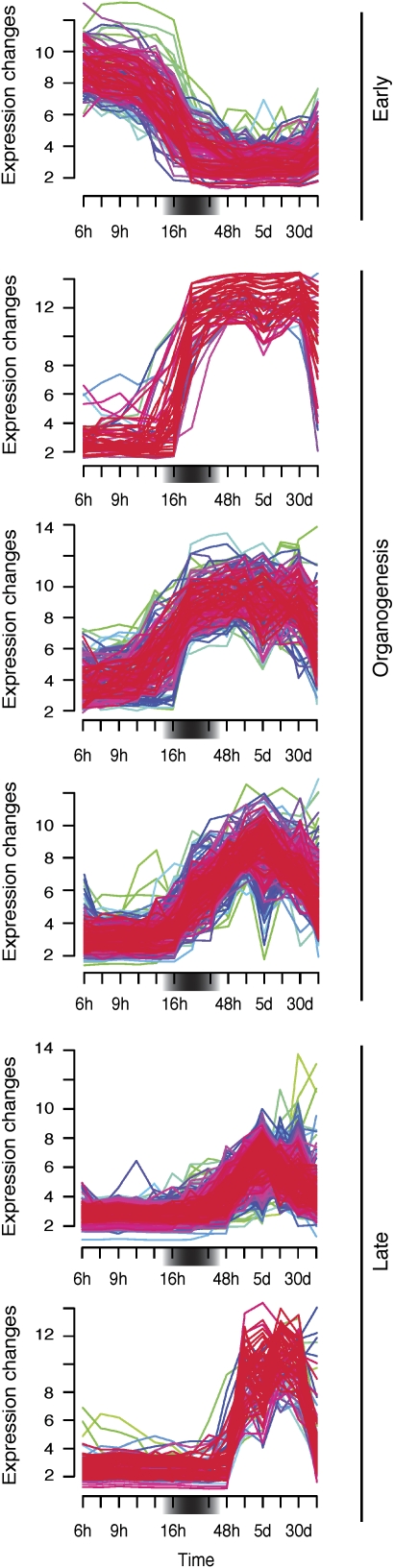
Expression profiles of “early,”“organogenesis,” and “late” genes. Each line represents a gene, color coded according to how well it is represented by the cluster, from yellow or green for low membership scores, to red or purple for high membership scores. The gray boxes on the *x*-axes indicate the presumed phylotypic period. All 25 clusters are presented in Fig. S1.

As an alternative to studying the expression profile of groups of candidate genes, we used soft clustering of expression profiles to generate groups of genes, whose properties may be related to the patterns of evolution and development (Fig. 6; Fig. S1). This provided us with three sets of genes with interesting profiles in development: (i) expression of the “early” genes is high early in development, and decreases to reach a stable low level by the presumed phylotypic period; (ii) expression of the “organogenesis” genes is low at early stages, then increases strongly at the presumed phylotypic period and remains high during larval development, with a decrease in adults; (iii) expression of the “late” genes is low both in early development and during the phylotypic period, with a later increase toward the larval stage.

The average number of abnormal phenotypes reported for mutation of genes from these groups differs significantly (*P*=0.0078, Kruskal–Wallis test). Mutation of “early” genes results in the most abnormal phenotypes (average of 10.5 vs. 5.28 for “organogenesis” genes and 6.86 for “late” genes). There is also a significant difference between the three categories for the number of anatomical structures in which each gene is detected (*P*=5.85E−11, Kruskal–Wallis test). This is mostly due to “late” genes being expressed in fewer structures (5.48 vs. 10.3 for “organogenesis” genes and 9.5 for “early” genes); in other words, “late” genes are more tissue-specific. As might be expected, expression of “early” genes is enriched in presumptive structures. Expression of “organogenesis” genes is enriched in numerous anatomical structures, most of them related to the nervous system, the visual system, the muscle, the heart, and the pancreas. And expression of “late” genes is enriched in the visual, intestinal, and nervous systems.

**Fig. 7:**
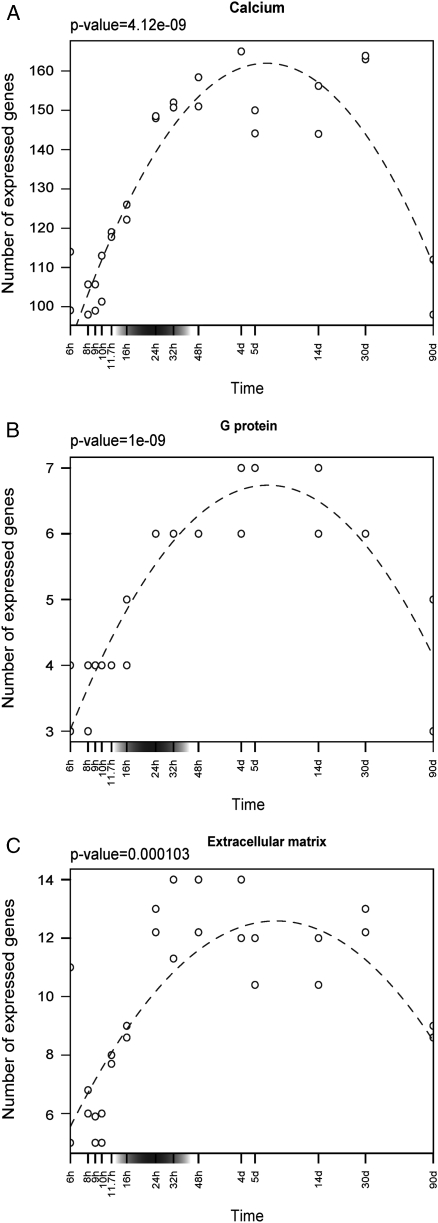
Variation of gene expression for genes involved in signaling in organogenesis. Number of expressed genes per developmental stage for (A) calcium (GO:0005262, GO:0019855, and GO:0005509; 196 genes); (B) heterotrimeric G protein complex (GO:0005578; seven genes); (C) proteinaceous extracellular matrix (GO:0005834; 18 genes). A polynomial model was fitted to the data (dashed line parabola) with *P*-values indicated above each plot. The gray boxes on the *x*-axes indicate the presumed phylotypic period. The *x*-axes are in logarithmic scale.

**Fig. 8:**
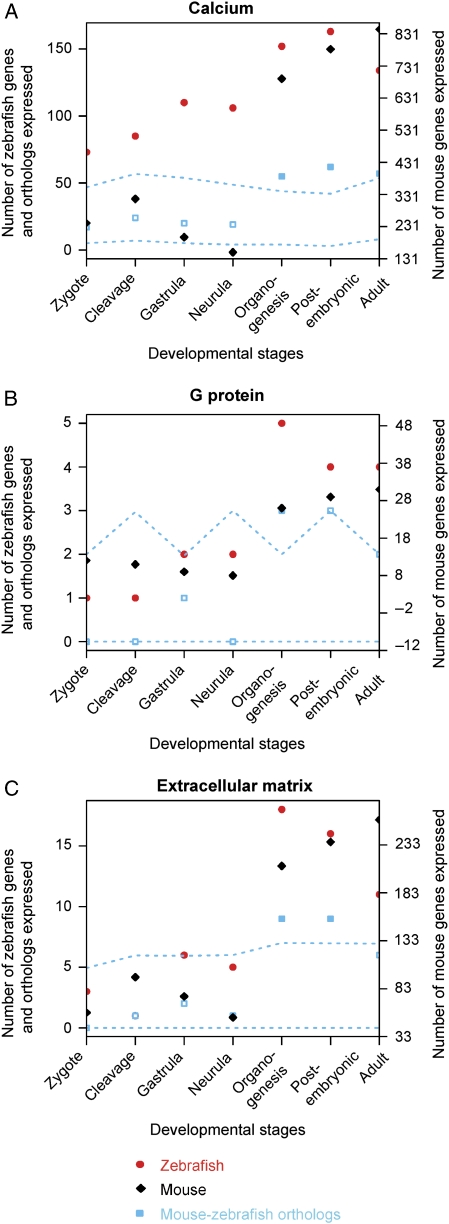
Conservation of gene expression for genes involved in signaling in organogenesis between zebrafish and mouse. Number of zebrafish (red circles) and mouse (black diamonds) genes, and ortholog pairs (blue squares) expressed per developmental stage for (A) calcium (GO:0005262, GO:0019855, and GO:0005509; 174 zebrafish and 862 mouse genes, 71 orthologs); (B) heterotrimeric G protein complex (GO:0005578; five zebrafish and 31 mouse genes, three orthologs); and (C) proteinaceous extracellular matrix (GO:0005834; 20 zebrafish and 265 mouse genes, 12 orthologs). The dotted lines represent the 1% confidence interval for conserved expression of orthologs; significant numbers of orthologs expressed are represented by filled squares. Organogenesis includes the presumed phylotypic period. The *x*-axis is not proportional to time, as the mapping of the stages of the two species compared on meta-stages is different. The scale of the *y*-axis is different for mouse, as more data are available.

An analysis of GO terms (Table 1) shows notably that “organogenesis” genes are enriched in proteins localized in the extracellular matrix, and in heterotrimeric G-protein complexes. This suggests a role in mediating cell or tissue interactions. Also of interest, these genes are enriched in molecular functions and biological processes related to calcium; calcium is a secondary messenger in many signal transduction pathways. However, calcium also plays a role in muscle contraction, and terms related to muscle are also enriched in “organogenesis” genes. It is difficult with our data to distinguish these two roles of calcium in development. Looking at the global pattern of genes from these GO categories, they have a similar expression profile to the signal transduction genes, with highest expression in larva (Fig. 7), and higher conservation of expression with mouse in organogenesis and postembryonic development (Fig. 8).

**Table 1:** **Gene ontology terms enriched or depleted according to expression profile in development**

Expression profile	GO^1^	Direction	GO ID	Term	Observed	Expected	*P*-value	Adjusted *P*-value (FDR)
Early	BP	Enriched	GO:0007368	Determination of left/right symmetry	9	0.41	6.40 E–11	4.90 E–8
			GO:0035050	Embryonic heart tube development	9	0.54	1.10 E–9	4.21 E–7
			GO:0007498	Mesoderm development	11	0.45	9.30 E–9	2.37 E–6
			GO:0001707	Mesoderm formation	5	0.25	2.40 E–6	4.60 E–4
			GO:0009953	Dorsal/ventral pattern formation	9	0.68	5.50 E–6	8.43 E–4
			GO:0030903	Notochord development	4	0.23	5.00 E–5	6.13 E–3
			GO:0040007	Growth	6	0.7	5.60 E–5	6.13 E–3
			GO:0042664	Negative regulation of endodermal cell fate specification	3	0.12	1.60 E–4	1.53 E–2
			GO:0001706	Endoderm formation	3	0.14	2.80 E–4	2.38 E–2
			GO:0009798	Axis specification	3	0.19	6.60 E–4	4.21 E–2
			GO:0045893	Positive regulation of transcription, DNA-dependent	3	0.19	6.60 E–4	4.21 E–2
			GO:0048264	Determination of ventral identity	3	0.19	6.60 E–4	4.21 E–2
	MF	Enriched	GO:0003700	Transcription factor activity	19	6.24	8.60 E–6	3.21 E–3
			GO:0008083	Growth factor activity	6	0.65	3.50 E–5	6.53 E–3
			GO:0043565	Sequence-specific DNA binding	14	4.97	3.50 E–4	4.35 E–2
	CC	Enriched	GO:0005634	Nucleus	28	13.11	3.70 E–5	5.74 E–3
Organogenesis	BP	Enriched	GO:0006816	Calcium ion transport	24	4.93	3.30 E–11	2.53 E–8
			GO:0006096	Glycolysis	9	1.44	6.20 E–6	2.37 E–3
			GO:0030239	Myofibril assembly	5	0.41	1.70 E–5	4.34 E–3
			GO:0015671	Oxygen transport	5	0.62	2.00 E–4	3.32 E–2
			GO:0051258	Protein polymerization	6	0.98	2.60 E–4	3.32 E–2
			GO:0006813	Potassium ion transport	6	0.98	2.60 E–4	3.32 E–2
	MF	Enriched	GO:0019855	Calcium channel inhibitor activity	22	5.05	2.30 E–9	5.22 E–7
			GO:0005262	Calcium channel activity	22	5.11	2.80 E–9	5.22 E–7
			GO:0005509	Calcium ion binding	28	8.26	6.10 E–9	7.58 E–7
			GO:0015662	ATPase activity, coupled to transmembrane movement of ions, phosphorylative mechanism	8	1.14	7.50 E–6	6.99 E–4
			GO:0030955	Potassium ion binding	5	0.49	4.80 E–5	2.98 E–3
			GO:0019870	Potassium channel inhibitor activity	5	0.49	4.80 E–5	2.98 E–3
			GO:0019825	Oxygen binding	5	0.65	2.60 E–4	1.39 E–2
			GO:0005267	Potassium channel activity	5	0.76	6.10 E–4	2.78 E–2
			GO:0015077	Monovalent inorganic cation transmembrane transporter activity	9	2.5	6.70 E–4	2.78 E–2
	CC	Enriched	GO:0016459	Myosin complex	5	0.46	3.60 E–5	5.35 E–3
			GO:0005882	Intermediate filament	5	0.51	6.90 E–5	5.35 E–3
			GO:0005833	Hemoglobin complex	5	0.56	1.20 E–4	6.20 E–3
			GO:0005856	Cytoskeleton	21	4.61	5.70 E–4	2.21 E–2
			GO:0005578	Proteinaceous extracellular matrix	6	1.18	8.20 E–4	2.54 E–2
			GO:0005834	Heterotrimeric G-protein complex	3	0.26	1.23 E–3	3.18 E–2
Late	BP	Enriched	GO:0006879	Cellular iron ion homeostasis	15	3.05	1.80 E–7	6.89 E–5
			GO:0006826	Iron ion transport	15	3.05	1.80 E–7	6.89 E–5
			GO:0006508	Proteolysis	22	7.2	1.90 E–6	4.85 E–4
			GO:0006783	Heme biosynthetic process	11	2.1	4.30 E–6	7.05 E–4
			GO:0007602	Phototransduction	5	0.33	4.60 E–6	7.05 E–4
			GO:0018298	Protein-chromophore linkage	5	0.38	1.20 E–5	1.53 E–3
			GO:0007601	Visual perception	10	1	3.00 E–4	3.28 E–2
	MF	Enriched	GO:0020037	Heme binding	13	2.48	5.80 E–7	2.16 E–4
			GO:0005506	Iron ion binding	16	4.45	6.30 E–6	1.17 E–3
			GO:0009881	Photoreceptor activity	4	0.23	2.30 E–5	2.61 E–3
			GO:0004252	Serine-type endopeptidase activity	8	1.31	2.80 E–5	2.61 E–3
			GO:0004866	Endopeptidase inhibitor activity	14	4.5	1.30 E–4	9.70 E–3
			GO:0004182	Carboxypeptidase A activity	4	0.42	4.90 E–4	3.05 E–2
			GO:0003746	Translation elongation factor activity	4	0.47	7.90 E–4	3.90 E–2
			GO:0008061	Chitin binding	3	0.23	9.40 E–4	3.90 E–2
			GO:0008533	Astacin activity	3	0.23	9.40 E–4	3.90 E–2
	MF	Depleted	GO:0003676	Nucleic acid binding	20	40.18	7.80 E–5	2.91 E–2
	CC	Enriched	GO:0005576	Extracellular region	15	5.31	2.10 E–4	3.26 E–2

1 GO ontologies: BP, biological process; MF, molecular function; CC, cellular component.

## DISCUSSION

On the basis of Raff's (1996) hypothesis that the conserved morphology between vertebrate species at the phylotypic period could be the result of specific interactions, we investigated different molecular aspects related to interactions and signaling during zebrafish development. It should be noted that the data available do not allow us to test directly the hypothesis about differences in modularity between developmental stages. We can only evaluate the overall importance of molecular interactions and signaling, not whether it occurs inside or among “modules.” But our working hypothesis is that major changes in signaling will probably affect the extent to which different regulatory mechanisms are used. Thus if the phylotypic period is defined by a specific pattern of interactions, we expect this period to be characterized by a specific signature of expression of genes involved in signaling and regulation.

A first notable observation is that many measures of signaling do present a peak during development (Figs. 3 and 7), and that these peaks seem to be evolutionarily conserved because they are also detected in mouse (Figs. 4 and 8). This stands in contrast to the monotonous decrease we previously reported for evolutionary constraints on the genome (Roux and Robinson-Rechavi 2008), and which is also observed for PPI centrality (Figs. 1 and 2). The other notable observation is that the peak rarely corresponds to the morphologically defined phylotypic period.

The only feature which peaks close to the phylotypic period is the number of transcription genes expressed (Fig. 3D). Combined with the onset of expression of a first wave of miRNAs (Fig. 5), this could be seen as supportive of strong regulation of gene expression during this period. But these and other features which increase during the phylotypic period do not decrease until much later; most present maxima during larval development (Figs. 3 and 7). There are, for example, more miRNAs expressed after than during the phylotypic stage, which is indicative of tight regulation of gene expression in late development. Moreover, when we classify genes according to their pattern of expression during development, there is no class of genes, which peak specifically during the phylotypic period, but rather many genes that increase during that period, then do not decrease significantly until adulthood (Fig. 6). These “organogenesis” genes are enriched in proteins with a potential role in signaling between cells or tissues, considering their cellular localization and their relation with calcium. In zebrafish, intracellular as well as localized and long-range intercellular calcium signaling patterns have been observed from cleavage to segmentation (Webb and Miller 2007). These calcium signaling events have been shown to be involved in dorso-ventral and left–right patterning, convergent extension during gastrulation and somite formation. A role for calcium signaling in development is not restricted to zebrafish, as experiments have also implicated calcium in dorso-ventral patterning and convergent extension movement as well as neural induction in *Xenopus*, in left–right patterning in mouse and chicken, and in somite formation in chicken (Whitaker 2006; Freisinger et al. 2008;). Indeed, the expression of calcium signaling genes in organogenesis and larval stages is conserved between zebrafish and mouse (Fig. 8A).

The late peak in the number of signal transduction and receptor genes expressed suggests a major role for cell, tissue, and receptor–ligand interactions. At the same time the majority of miRNAs are expressed at a high level and consequently mediate numerous RNA–RNA interactions. This probably reflects the increasing complexity of the organism, and the need for specific regulation in differentiated organs and tissues. This specialization is supported by the tissue specificity of “late” genes.

While the separation between a phylotypic period and further organogenesis and larval development is thus not clearly defined by any type of gene expression, early development does present a quite specific pattern. This can be seen e.g. in the conservation of gene coexpression between zebrafish and mouse: whereas the conservation of coexpression of interacting proteins is highest in early development (Fig. 2), conservation of signaling gene expression is lowest (Fig. 4). Moreover, we can identify a cluster of 160 genes that are highly expressed early in development, but have practically lost expression by pharyngula (24 h), and remain at very low levels thereafter (Fig. 6). These specific “early” genes are enriched in terms related to body plan specification (Table 1). Thus the information for the body plan appears to be laid out before the phylotypic period, when genes are under the strongest evolutionary constraints (Roux and Robinson-Rechavi 2008). The observation that mutation of these “early” genes produces the most diverse abnormal phenotypes is also consistent with a key role for early development, rather than for the phylotypic period. These early genes appear to participate highly in conserved PPI (Figs. 1 and 2), whereas miRNA regulation is almost absent (Fig. 5; Wienholds et al. 2005). This pattern is inversed from organogenesis to larval development (high miRNA regulation, small role of PPI).

These results pose the question of why a phylotypic period is observed at the morphological level. True, there are many molecular interactions around that period of zebrafish development, and they seem to be conserved with mouse. But they mostly continue into further organogenesis and larval development, sometimes even reaching a maximum during the larval stage, which is not morphologically conserved. We suggest that a solution lies in realizing that morphology at each stage of development probably depends on an interaction between morphology at the previous stage and the genes expressed, which act to modify this morphology (Richardson 1999). Under this simple assumption, early development would be constrained by its starting point, that is, the very divergent zygotic morphologies (Raff 1996; Solnica-Krezel 2005;); under the influence of the conserved genetic determinants of early development (Roux and Robinson-Rechavi 2008), morphology should tend to converge (also suggested for insects Cruickshank and Wade 2008); and finally the rapidly evolving genes expressed in later development should cause a corresponding divergence in morphology. This explanation allows for a minimum in morphological divergence at mid development, without any corresponding peak in genetic or molecular processes.

## CONCLUSION

There are high levels of interactions between molecules, and between cells and tissues, during the presumed phylotypic period, conserved between zebrafish and mouse. But there does not appear to be a marked boundary in levels or types of interactions, nor in zebrafish–mouse conservation, between that period and later development, where morphology is more divergent between species. On the other hand, expression and interaction data show a marked change between early (prephylotypic period) and later development. Early expressed genes appear to be both more conserved between zebrafish and mouse, and regulated by different pathways, than other genes, with more PPI and little or no miRNA regulation. We propose that morphological conservation at the phylotypic period is a consequence of this early genetic conservation.

## SUPPORTING INFORMATION

**Fig. S1.** Gene clustering according to expression in development. Twenty-five clusters of genes obtained by soft clustering. Cluster 15 corresponds to the “early” genes. Clusters 1, 20 and 23 correspond to the “organogenesis” genes. Clusters 3 and 8 correspond to the “late” genes. Soft clustering assigns a gene gradual degrees of membership to a cluster. The membership scores indicate how well the gene is represented by a cluster, and are color-coded from yellow or green for low membership scores to red or purple for high membership scores. The gray boxes on the x-axes indicate the presumed phylotypic period.

**Fig. S2.** miRNA clustering according to expression in development. Two clusters of miRNA obtained by soft clustering. Soft clustering assigns a miRNA gradual degrees of membership to a cluster. The membership scores indicate how well the miRNA is represented by a cluster, and are color-coded from yellow or green for low membership scores to red or purple for high membership scores. The gray boxes on the x-axes indicate the presumed phylotypic period.

**Fig. S3.** Variation of miRNA target genes expression during development. Difference in median gene expression between targets of “early onset” and “late onset” miRNAs. The dashed lines represent the 5% confidence interval; significant differences are represented by filled circles. The gray box on the x-axis indicates the presumed phylotypic period. The x-axis is in logarithmic scale.

**Fig. S4.** Variation of centrality in the protein-protein interaction network for signal transduction genes during development. Same as Fig. 1, but restricted to the gene categories used in Fig. 3.

**Fig. S5.** Variation of centrality in the protein-protein interaction network for non-signal transduction genes during development. Same as Fig. 1, but restricted to the genes that do not belong to any of the categories used in Fig. 3 (*n*=7399).
