# Supplementary material for: Molecular signaling in zebrafish development and the vertebrate phylotypic period
Source: Evol Dev. 2010 Mar;12(2):144–56. doi: 10.1111/j.1525-142X.2010.00400.x (PMC2855863; doi:10.1111/j.1525-142X.2010.00400.x)
Supplement: Supplementary file 1 [file ede0012-0144-SD1.pdf]

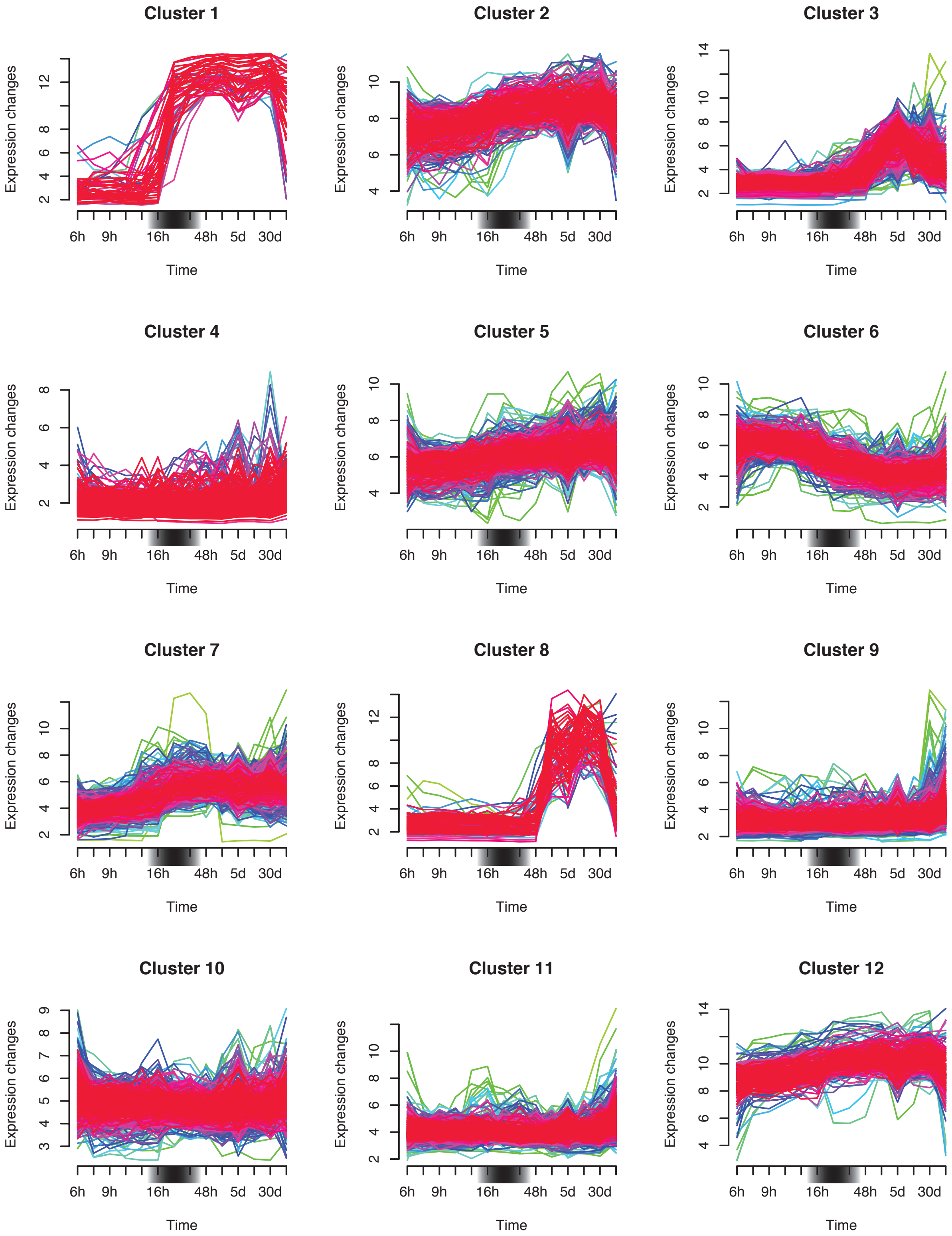

Cluster 1
Expression changes
2 4 6 8 12
6h 9h 16h 48h 5d 30d
Time
Cluster 2
Expression changes
4 6 8 10
6h 9h 16h 48h 5d 30d
Time
Cluster 3
Expression changes
2 4 6 8 10 14
6h 9h 16h 48h 5d 30d
Time
Cluster 4
Expression changes
2 4 6 8
6h 9h 16h 48h 5d 30d
Time
Cluster 5
Expression changes
4 6 8 10
6h 9h 16h 48h 5d 30d
Time
Cluster 6
Expression changes
2 4 6 8 10
6h 9h 16h 48h 5d 30d
Time
Cluster 7
Expression changes
2 4 6 8 10
6h 9h 16h 48h 5d 30d
Time
Cluster 8
Expression changes
2 4 6 8 12
6h 9h 16h 48h 5d 30d
Time
Cluster 9
Expression changes
2 4 6 8 10
6h 9h 16h 48h 5d 30d
Time
Cluster 10
Expression changes
3 4 5 6 7 8 9
6h 9h 16h 48h 5d 30d
Time
Cluster 11
Expression changes
2 4 6 8 10
6h 9h 16h 48h 5d 30d
Time
Cluster 12
Expression changes
4 6 8 10 14
6h 9h 16h 48h 5d 30d
Time

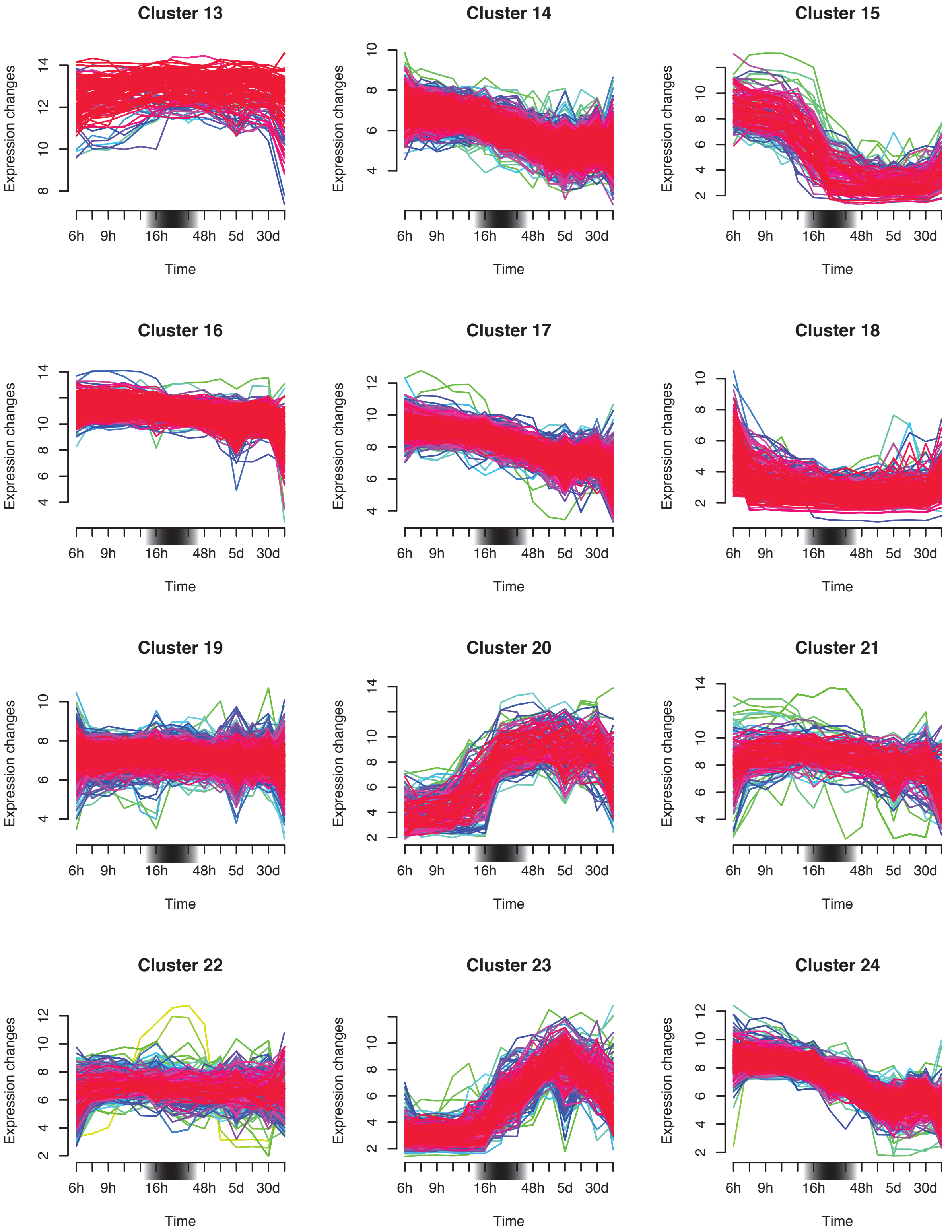

Cluster 13
Cluster 14
Cluster 15
Cluster 16
Cluster 17
Cluster 18
Cluster 19
Cluster 20
Cluster 21
Cluster 22
Cluster 23
Cluster 24
Expression changes
Time
6h
9h
16h
48h
5d
30d

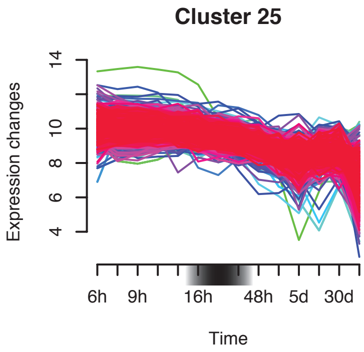

Cluster 25
Expression changes
4
6
8
10
14
6h
9h
16h
48h
5d
30d
Time

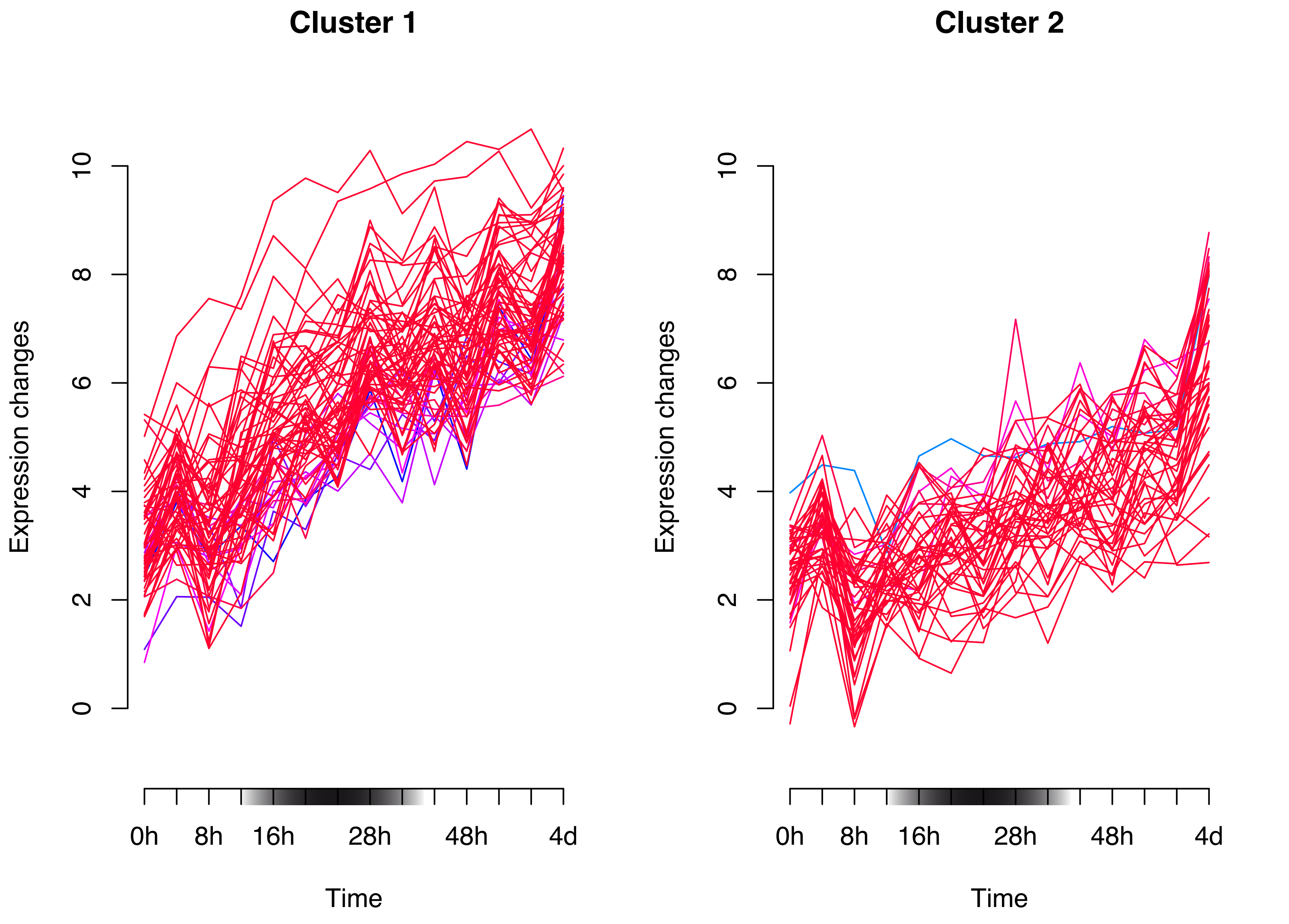
Cluster 1
Cluster 2
Expression changes
Expression changes
0
2
4
6
8
10
0
2
4
6
8
10
0h
8h
16h
28h
48h
4d
0h
8h
16h
28h
48h
4d
Time
Time

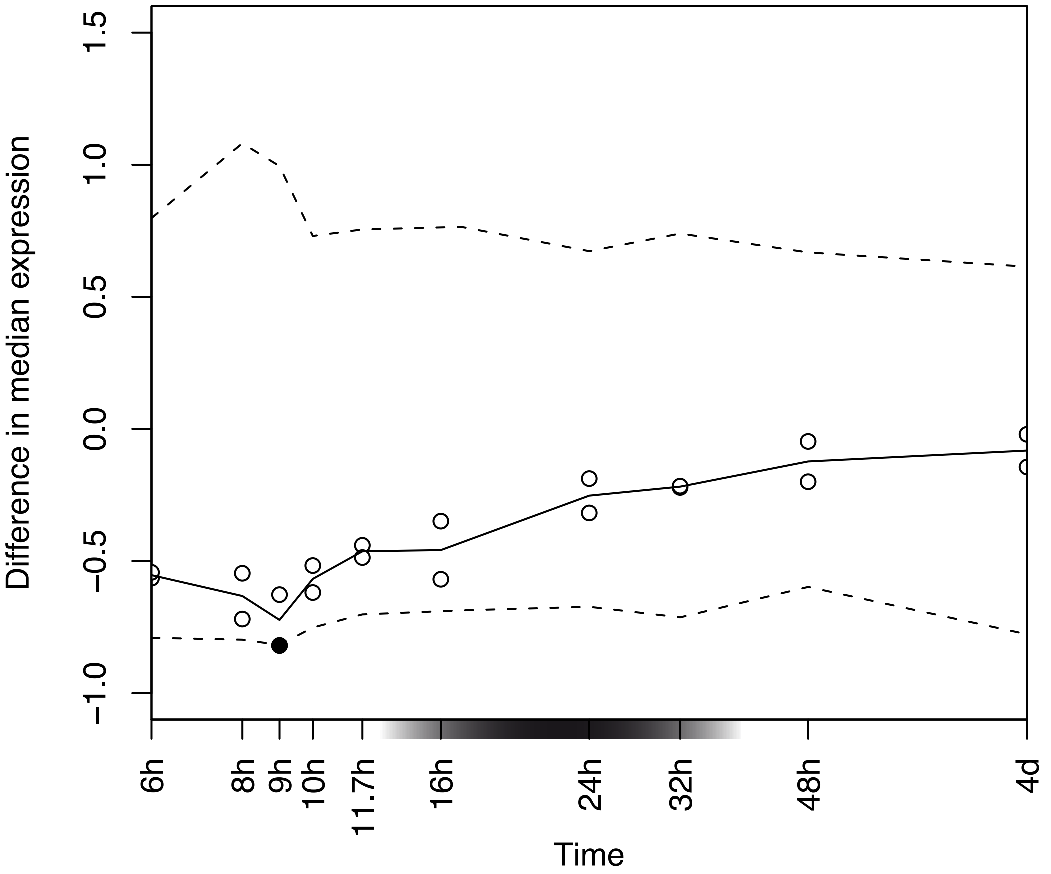

Difference in median expression
1.5
1.0
0.5
0.0
−0.5
−1.0
6h
8h
9h
10h
11.7h
16h
24h
32h
48h
4d
Time

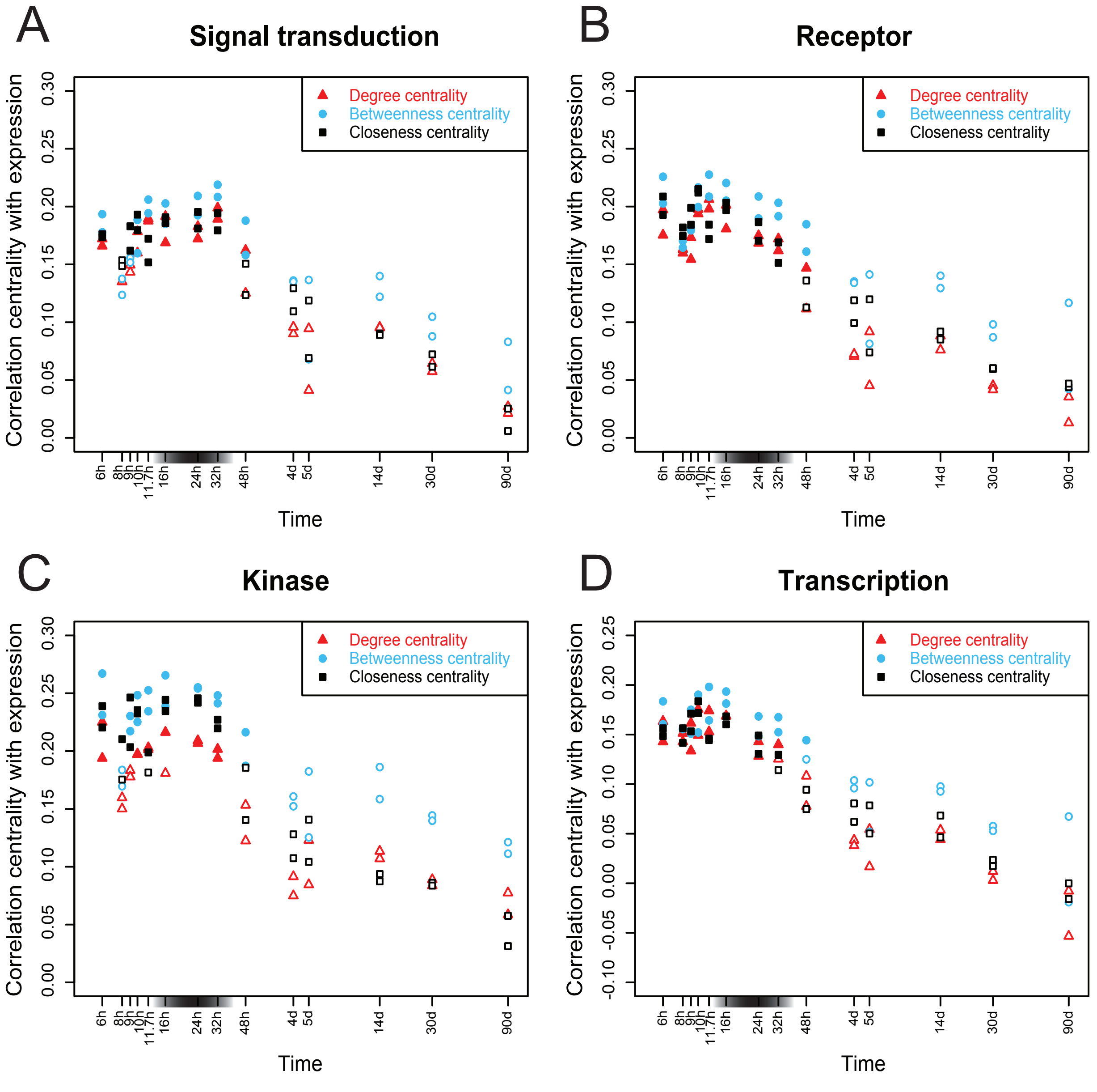

A
Signal transduction
B
Receptor
C
Kinase
D
Transcription
Degree centrality
Betweenness centrality
Closeness centrality
Correlation centrality with expression
Time
6h
8h
9h
10h
11.7h
16h
24h
32h
48h
4d
5d
14d
30d
90d
0.00
0.05
0.10
0.15
0.20
0.25
0.30
-0.10
-0.05

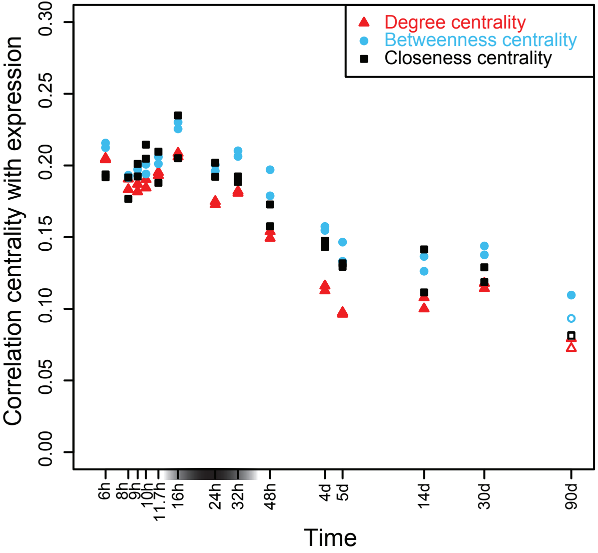

Degree centrality
Betweenness centrality
Closeness centrality
Correlation centrality with expression
0.00
0.05
0.10
0.15
0.20
0.25
0.30
6h
8h
9h
10h
11.7h
16h
24h
32h
48h
4d
5d
14d
30d
90d
Time
